# Novel insights into the host immune response of chicken Harderian gland tissue during Newcastle disease virus infection and heat treatment

**DOI:** 10.1186/s12917-018-1583-0

**Published:** 2018-09-12

**Authors:** Perot Saelao, Ying Wang, Rodrigo A. Gallardo, Susan J. Lamont, Jack M. Dekkers, Terra Kelly, Huaijun Zhou

**Affiliations:** 1 Integrative Genetics and Genomics Graduate Group, University of California, Davis, CA 95616 USA; 2 Genomics to Improve Poultry Innovation Lab, University of California, Davis, CA 95616 USA; 3 Department of Animal Science, University of California, Davis, CA 95616 USA; 4 School of Veterinary Medicine, University of California, Davis, CA 95616 USA; 5 Department of Animal Science, Iowa State University, Ames, IA 50011 USA; 6 One Health Institute, University of California, Davis, CA 95616 USA

**Keywords:** Newcastle disease virus, Heat stress, Harderian gland, RNA-Seq, Disease resistance

## Abstract

**Background:**

Newcastle disease virus, in its most pathogenic form, threatens the livelihood of rural poultry farmers where there is a limited infrastructure and service for vaccinations to prevent outbreaks of the virus. Previously reported studies on the host response to Newcastle disease in chickens have not examined the disease under abiotic stressors, such as heat, which commonly experienced by chickens in regions such as Africa. The objective of this study was to elucidate the underlying biological mechanisms that contribute to disease resistance in chickens to the Newcastle disease virus while under the effects of heat stress.

**Results:**

Differential gene expression analysis identified genes differentially expressed between treated and non-treated birds across three time points (2, 6, and 10 days post-infection) in Fayoumi and Leghorn birds. Across the three time points, Fayoumi had very few genes differentially expressed between treated and non-treated groups at 2 and 6 days post-infection. However, 202 genes were differentially expressed at 10 days post-infection. Alternatively, Leghorn had very few genes differentially expressed at 2 and 10 days post-infection but had 167 differentially expressed genes at 6 days post-infection. Very few differentially expressed genes were shared between the two genetic lines, and pathway analysis found unique signaling pathways specific to each genetic line. Fayoumi had significantly lower viral load, higher viral clearance, higher anti-NDV antibody levels, and fewer viral transcripts detected compared to Leghorns. Fayoumis activated immune related pathways including SAPK/JNK and p38 MAPK signaling pathways at earlier time points, while Leghorn would activate these same pathways at a later time. Further analysis revealed activation of the GP6 signaling pathway that may be responsible for the susceptible Leghorn response.

**Conclusions:**

The findings in this study confirmed our hypothesis that the Fayoumi line was more resistant to Newcastle disease virus infection compared to the Leghorn line. Within line and interaction analysis demonstrated substantial differences in response patterns between the two genetic lines that was not observed from the within line contrasts. This study has provided novel insights into the transcriptome response of the Harderian gland tissue during Newcastle disease virus infection while under heat stress utilizing a unique resistant and susceptible model.

**Electronic supplementary material:**

The online version of this article (10.1186/s12917-018-1583-0) contains supplementary material, which is available to authorized users.

## Background

Newcastle disease virus (NDV) is a negative-sense, single stranded RNA virus in the family *Paramyxoviridae* that infects a wide range of avian species. There exist several different strains of the virus, each defined by their pathogenicity and grouped as: asymptomatic, lentogenic (nonvirulent), mesogenic (intermediate virulence), and velogenic (highly virulent) [1]. Outbreaks of virulent strains of NDV in poultry farms can result in 80–90% mortality [2]. Globally, the virus represents a major threat to food security in rural areas, and represents a huge economic drain during outbreaks [3]. Although vaccines exist for NDV, the lack of infrastructure and “cold chain” in under-developed countries limits the protection that vaccination can offer to address Newcastle disease. Genetic improvement of disease resistance provides an alternative approach to further reduce the likelihood of Newcastle disease outbreaks in less developed countries.

In addition to the threat of biotic factors, abiotic factors such as heat stress threaten have become one of the most economically devastating factors for poultry farmers. The overall impact of heat stress on poultry flocks is estimated to result in a loss of $165 millions dollars annually to the poultry industry in the U.S. [4]. Heat stress is characterized as the result of a net energy imbalance between an organism’s body and its environment [5]. This energy in the form of heat is unable to dissipate into the environment and thus accumulates in the host resulting in high internal temperatures that cause a dysregulation of neuroendocrine, behavioral, and metabolic systems [5]. In chickens, this physiological impairment can result in an overall decrease in production quality traits such as egg yield, egg quality, body weight, and reduced immune function [6, 7]. Bartlett and Smith reported that heat stress reduced the total level of circulating IgM and IgG antibodies during primary and secondary immune response [7]. In HD11 cell lines however, Slawinska et al. found that heat stress of LPS treated cell lines resulted in an up regulation of some immune related genes potentially due to the increase abundance of heat shock proteins and chaperones [8]. A few studies have suggested that host genetic makeup plays a significant role in response to heat stress in chickens [9, 10]. The increasing impact of climate change on global temperatures necessitates a greater emphasis on understanding the role of abiotic factors have on host physiology and immune response.

To develop novel methods to limit economic losses due to biotic and abiotic stress factors in poultry, it is essential to gain a deeper understanding of the immune response elicited under the simultaneous effects of these two factors. Transcriptome profiling offers the potential to gain insight into the host’s entire gene expression profile and the complex biological processes underlying the host immune response. Several studies on the transcriptome of the chicken immune system during NDV infection have focused on the trachea, lung, and spleen [11–13]. Nuss et al. highlighted the importance of tissue-specific expression profiling in understanding infection specific functions regulating the host-pathogen transcriptome [14]. In chickens, the Harderian gland is a key immune organ and major site of infection for NDV due to its proximity to the eye. The Harderian gland is located within the inner orbit of the eye and functions as a major component of the head associated lymphoid tissues (HALT) containing secretory bodies of antibodies and other immune cells [15]. In addition to its function as an immune organ, the Harderian gland also plays a role in thermoregulation and the production of thermal regulator secretions [15]. Despite its critical role as an essential immune organ, very few studies have attempted to profile the Harderian gland specific host response to pathogen infection at the transcriptome level.

The genetic makeup of the host has a significant impact on disease resistance and heat tolerance. Previous research in chickens has investigated the genetics of resistance to pathogens by utilizing two highly inbred chicken lines, Fayoumi and Leghorn. The Fayoumi line has been used to understand relative resistance to a wide range of pathogens including, avian influenza virus (AIV), Marek’s disease virus, coccidiosis, and *Salmonella* [16–19]. Wang et al. demonstrated that the Fayoumi line was relatively more resistant to AIV when compared to Leghorns, with Fayoumis having a reduced AIV viral titer and an increase in signaling pathways associated with immune function [16]. Furthermore, Fayoumi is believed to be relatively more heat tolerant when compared to Leghorn based on blood chemistry analysis comparing the two genetic lines [20].

We hypothesized that Fayoumi birds were more NDV resistant while under the effects of heat stress relative to Leghorn. The specific objective of this study was to profile the host immune response to NDV infection under heat stress, and to identify genes and signaling pathways associated with disease resistance of the Harderian gland transcriptome at three time post-infection points during NDV infection and heat stress in both Fayoumi and Leghorn.

## Results

### The Fayoumi line was more resistant to NDV than leghorns while under heat stress

**Fig. 1:**
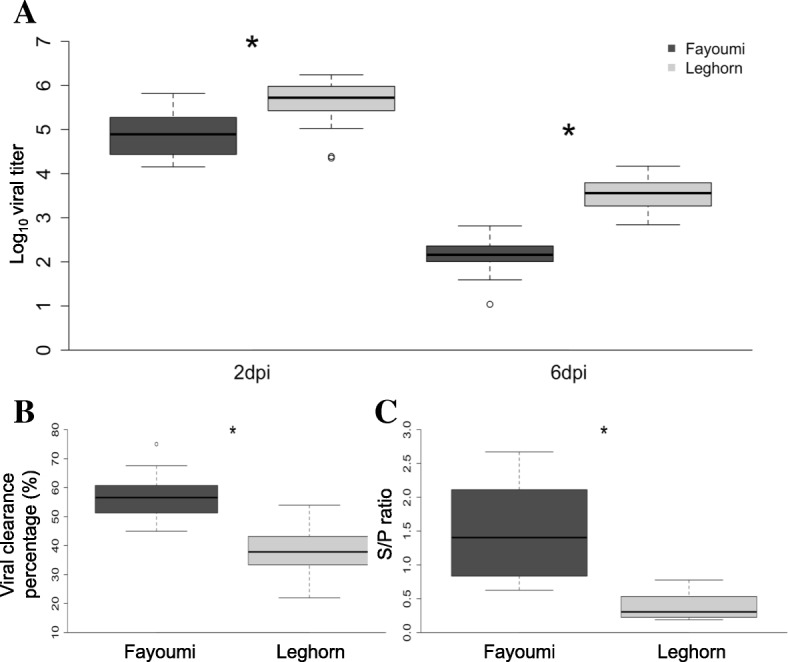
Box plots for viral titer, viral clearance, and anti-NDV antibody level of Fayoumi and Leghorn birds by days post-infection. Significance (*p* < 0.05) of the differences is indicated by *. **a** Log_10_ viral titer measured by qPCR in Fayoumi and Leghorn birds at 2 dpi (*p* = 1.23E-05) and 6 dpi (*p* = 1.46E-10). **b** Viral clearance rate of Fayoumi and Leghorn birds measured as the percent change in viral load from 2 to 6 dpi (*p* = 3.57E-07). **c** Anti-NDV antibody S/P ratio at 10 dpi (*p* = 1.5E-03)

NDV viral load was measured by qRT-PCR from extracted chicken lachrymal fluid determined that Fayoumi chickens had significantly lower NDV titers than Leghorns at both 2 and 6 dpi (*p* = 1.23E-05, *p* = 1.46E-10, respectively, Fig. 1). In addition, there was a significant difference in virus clearance rate from 2 to 6 dpi between the two lines (*p* = 3.57E-07). On average, the Fayoumi chickens line were able to clear 56% of the virus from 2 to 6 dpi, while the Leghorn line only cleared 37% of the virus from 2 to 6 dpi. Furthermore, anti-NDV antibody titers measured by ELISA in 10 dpi serum samples revealed that Fayoumis produced significantly more anti-NDV antibodies than Leghorn birds (*p* = 1.5E-03).

### Viral genome alignment identifies less NDV gene expression at the site of infection in Fayoumi than leghorn

**Fig. 2:**
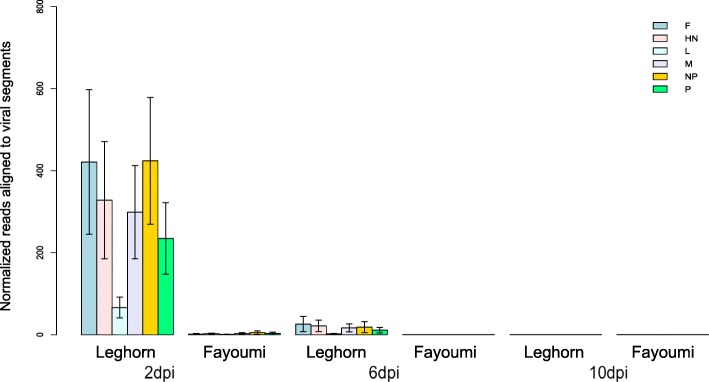
Normalized number of reads that aligned to the La Sota viral genome and to specific gene segments: Fusion glycoprotein (F), Hemagglutinin-neuraminidase (HN), RNA-directed RNA polymerase L (L), Matrix protein (M), Nucleoprotein (NP), and Phophoprotein (P). Reads were extracted from treated individuals by genetic line and time point and aligned to the NDV La Sota genome. Bars indicate normalized number of reads with standard error. A higher number of reads aligned to the viral genome in Leghorn at 2 dpi and 6 dpi, with no reads detectable in either line at 10 dpi

Viral transcripts extracted from the Harderian gland transcriptome sequences of NDV-infected individuals from both lines were aligned to the NDV La Sota strain genome. In Fayoumi chickens, NDV transcripts were only detected at 2 dpi and but not at 6 and 10 dpi, while Leghorn chickens had significantly higher quantities of the NDV transcripts detected at 2 dpi, with detectable quantities of the virus transcripts at 6 dpi and none at 10 (Fig. 2).

### Differential gene expression analysis within lines identified time specific responses to NDV infection under heat treatment

**Fig. 3:**
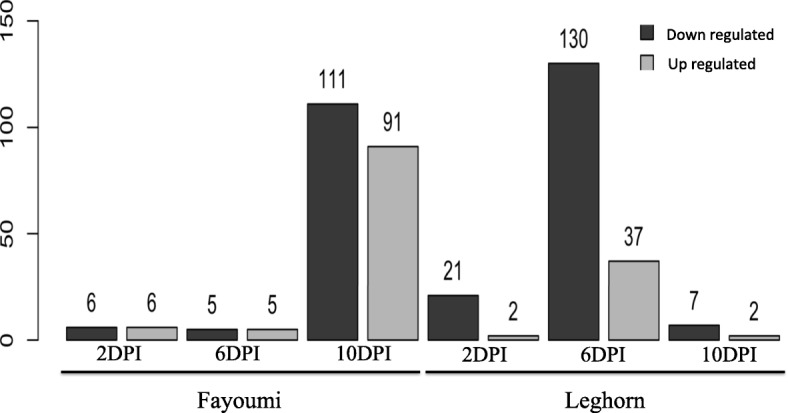
Number of differentially expressed genes identified between treated and non-treated birds by genetic line and time point. A false discovery rate < 0.05 was used to classify genes as differentially expressed. Genes are signified by color as up regulated (dark grey) or down regulated (light grey)

To understand the difference in the host gene expression response to the combined effect of NDV and heat stress within Fayoumis and Leghorns, contrasts were made comparing treated and non-treated birds across three time points (2, 6, and 10 dpi). In the Fayoumi line, only 12 and 10 genes were differentially expressed between treated and non-treated birds at 2 and 6 dpi, respectively (Fig. 3). However, at 10 dpi the overall number of differentially expressed genes (DEGs) substantially increased to 202 at 10 dpi, with 111 genes up regulated and 91 genes down regulated. Within the Leghorn line, 23 genes were differentially expressed at 2 dpi, with 21 genes up regulated (Fig. 3). However, at 6 dpi the total number of DEGs increased to 167, with 130 genes up regulated and 37 genes down regulated. Finally, at 10 dpi the number of DEGs decreased to only 9.

**Fig. 4:**
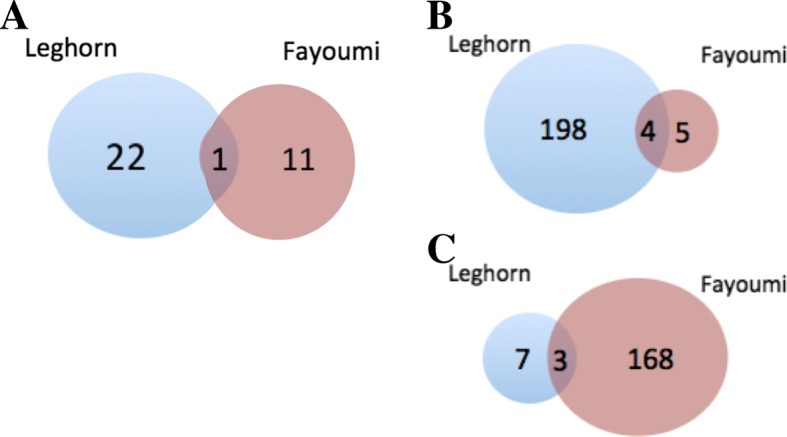
Venn diagrams displaying the number of overlapping differentially expressed genes that overlap between genetic line by time point when comparing treated vs. non-treated birds. **a** Overlapped DEGs between Fayoumi (22) and Leghorn (11) genes at 2 dpi. **b** Overlapped DEGs between the two genetic lines at 6 dpi and **c** Overlapped DEGs between the two genetic lines at 10 dpi

Comparison of DEGs identified in response to NDV and heat within line demonstrated very little overlap between Fayoumi and Leghorn. Across time points, 1, 4, and 3 DEGs were shared between the two lines at 2, 6, and 10 dpi, respectively (Fig. 4).

**Fig. 5:**
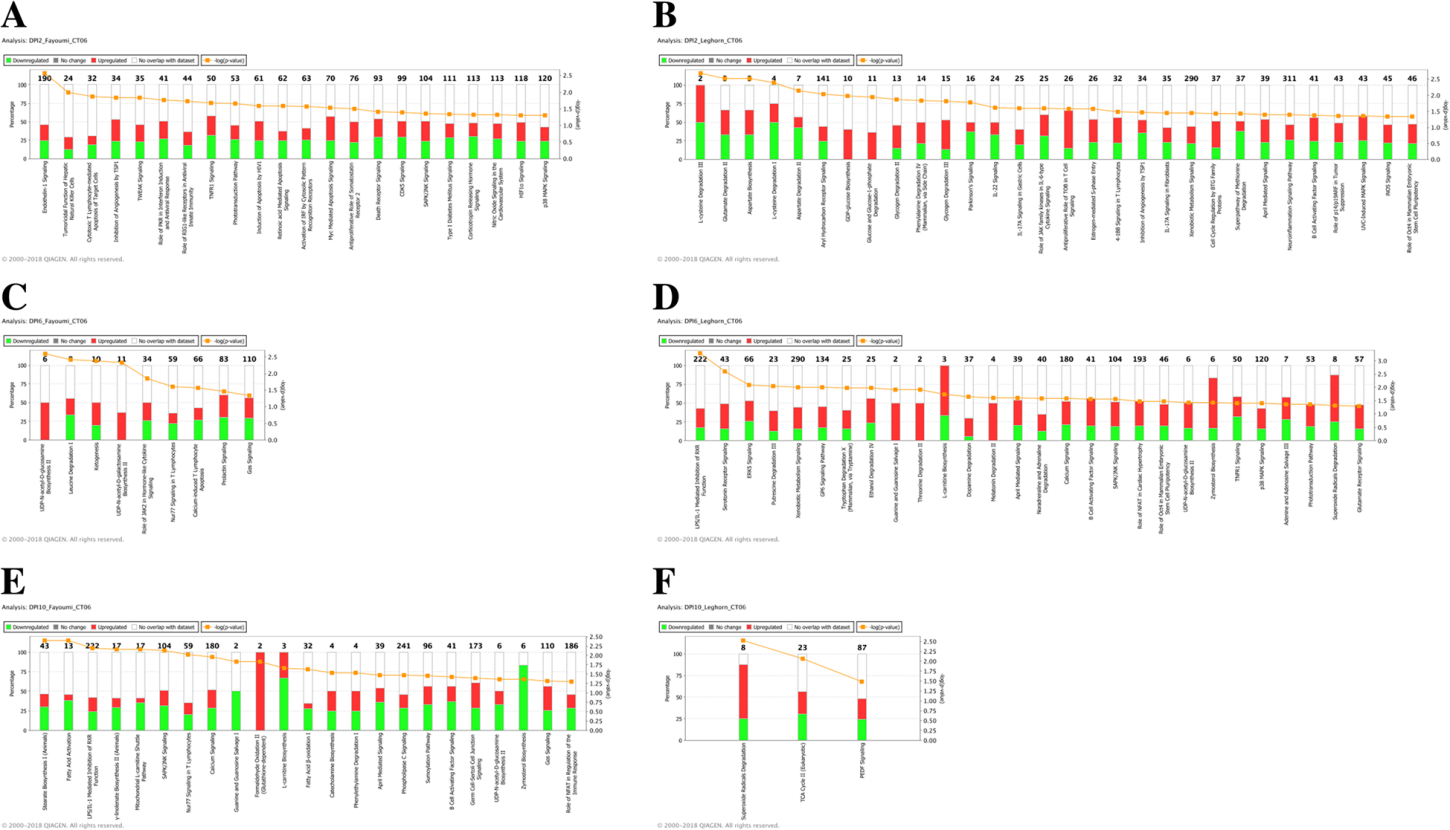
Significantly enriched pathways identified through Ingenuity Pathway Analysis among differentially expressed genes by dpi and genetic line. **a** Fayoumi at 2 dpi, **b** Leghorn at 2 dpi, **c** Fayoumi at 6 dpi, **d** Leghorn at 6 dpi, **e** Fayoumi at 10 dpi, and **f** Leghorn at 10 dpi. Figures identify the number of genes in each pathway shown in black, if the gene is up (red) or down (green) regulated in the pathway, and its significant –log(*p* value) shown in orange

Pathway analysis of the DEGs identified between treated and non-treated birds from within line contrasts was used to identify signaling pathways that were enriched at each time point. For Fayoumi at 2 dpi, 22 pathways were significantly enriched (Fig. 5a, c, e), of which 14 were associated with immune-related functions, which include pathways such as RIG1-like receptors in antiviral innate immunity, IRF activation, SAPK/JNK signaling, and p38 MAPK signaling. At 6 dpi, only 9 pathways were enriched between treated and non-treated Fayoumi birds, including immune pathways such as T lymphocyte activity and cytokine signaling were significantly different between treated and non-treated Fayoumi birds. At 10 dpi, there was a substantial increase in the total number of enriched pathways (23), with the majority of genes involved in these processes down regulated. Interestingly, pathways such as Nur77 signaling in T lymphocytes and gas signaling were significantly enriched across 6 and 10 dpi.

**Fig. 6:**
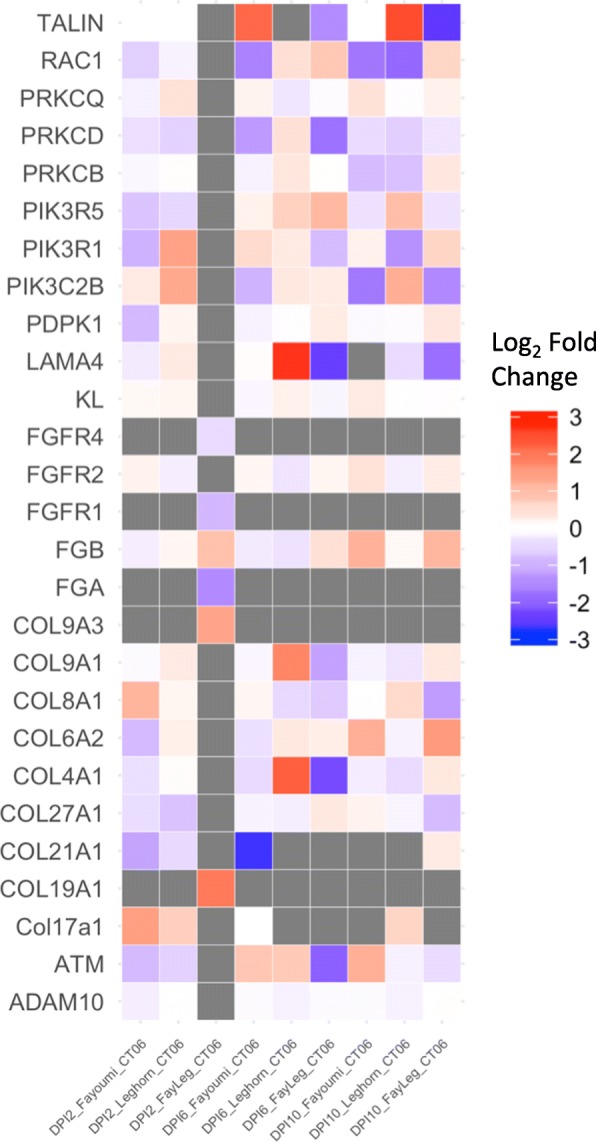
Heat map of the expression of genes involved with the GP6 signaling pathway for Fayoumi, Leghorn, and the interaction effect between lines across all three time points. Grey boxes signify no signal was detected of the gene at the specific comparison and time point

Similar to Fayoumi, pathway analysis was performed for DEGs identified between treated and non-treated Leghorns across all three time points (Fig. 5b, d, f). At 2 dpi, 30 enriched pathways were significantly enriched among DEGs. At 6 dpi, 28 pathways were significantly enriched, with GP6 signaling pathway having a positive activation z-score of 2.0. Two genes in this pathway, LAMA4 and COL4A1 were significantly up regulated in the treated compared to non-treated birds (Fig. 6). At 10 dpi, only three significant pathways were enriched among DEGs, which included superoxide radicals degradation, TCA cycle II, and PEDF signaling functions. The entire list of DEGs between treated and non-treated birds within each line and time point are included in Additional file 1.

### Interaction analysis of treatment by genetics revealed unique and line-specific response patterns to NDV during heat treatment

**Fig. 7:**
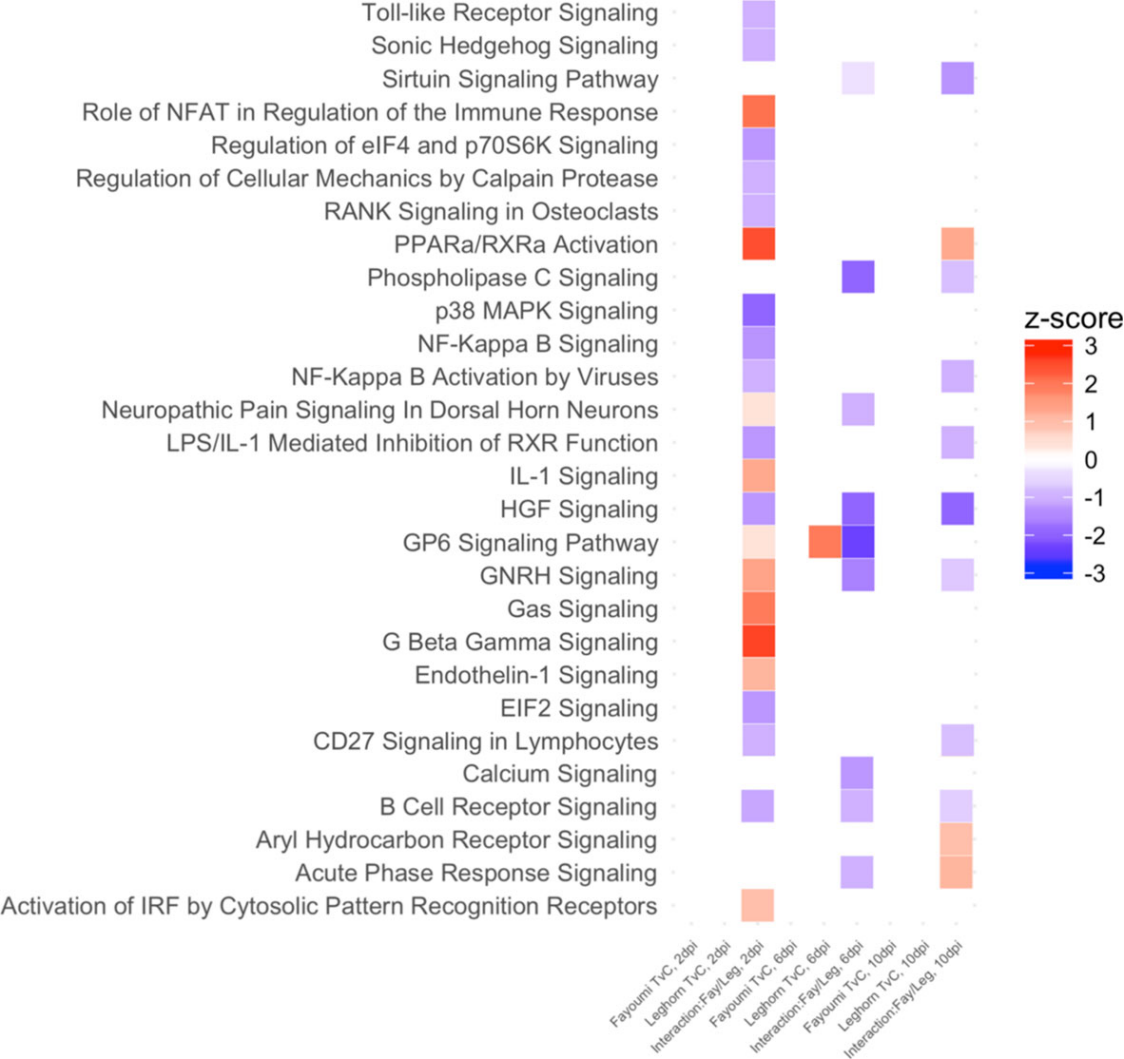
Heat map of predicted activation or inhibition of significant pathways from Ingenuity Pathway Analysis that were significantly (−log(*p* value) > 1.3) enriched and that had a Z-score > |1| for the effect of treatment within each genetic line and for the interaction effect between line and treatment for each time point. Complete names of the pathways show the predicted activation of the pathway (Z-score > 1) or inhibition of the pathways (Z-score < − 1). Predicted Z-score for the interaction effect identify pathways primarily enriched in Fayoumi (Z-score > 1) or Leghorn (Z-score < − 1)

While genes associated with NDV infection under heat stress within each line are important to understand the host immune response to pathogen infection, genes that differ between lines that are associated with resistance to NDV infection are more critical in elucidating the underlying mechanisms that contribute to differences between the Fayoumi and Leghorn lines. To identify such genes and signaling pathways, interaction analysis of treatment by line was conducted, resulting in 757, 194, and 403 DEGs for this interaction at 2, 6, and 10 dpi, respectively. Further, pathway analysis identified many pathways that were significantly enriched among these DEGs that were not found for the within line contrasts (Fig. 7). In total, 26 pathways were enriched at 2 dpi, with 15 enriched in Leghorn including NF-Kappa B signaling and activation, p38 MAPK, and IL-1 mediated inhibition of RXR function and 11 for Fayoumi including PPARa/RXRa activation and G beta gamma signaling, which had the highest absolute Z-score value of all significant pathways. At 6 dpi, all significant pathways identified were enriched in Leghorn that include: GP6 signaling, GNRH signaling, calcium signaling, acute phase response signaling, phospholipase C signaling, and sirtuin signaling. Finally at 10 dpi, 13 pathways were enriched with 8 enriched in Leghorns and 5 in Fayoumis. HGF signaling was the most significant pathway identified at 10 dpi, and it was the only pathway significant across all three time points in Leghorns. HGF signaling and its respective genes play a role in regulating apoptotic processes and activates STAT3 and PI-3 kinase activity to affect inflammation and other cell phenotypes [21].

## Discussion

The study presented is part of the Feed the Future Innovation Lab for Genomics to Improve Poultry program to further the body of knowledge regarding the genetic basis of disease resistance to NDV in chickens. Two parallel animal-pathogen challenge experiments using the same inbred lines (Fayoumi and Leghorn chickens) were conducted at the University of California, Davis (UCD) and Iowa State University (ISU). The study at ISU focused on the host response to NDV inoculation, while the study at UCD focused on the host response to NDV inoculation under heat stress, a condition commonly experienced by village poultry flocks Africa. In the present study, Fayoumi birds had significantly lower levels of NDV in the chicken lachrymal fluid at both 2 dpi and 6 dpi, along with a much higher viral clearance rate than Leghorn birds. Anti-NDV antibody titers were also significantly higher in Fayoumis than in Leghorns. These results were consistent with NDV expression in the Harderian gland at both time points (Fig. 2). Significant differences in viral load between the two lines were only observed at 6 dpi in previous studies comparing NDV infected Fayoumi and Leghorn birds without heat stress [11]. This result further suggests that environmental stress could significantly differentiate the host response to NDV infection between two genetic lines. We speculate that Fayoumis were able to have significantly lower viral titer levels than Leghorns at the initial stage of the infection. This difference suggests either that Fayoumi may be mounting a more robust early immune response while under the effects of heat stress, or that the heat treatment has significantly impacted Leghorns’ ability to respond the infection as effectively as Fayoumis. Other studies in avian influenza have indicated that heat stress could reduce the viral load of the host [22]. Activation of heat stress proteins could play a protective role during virus infection [23, 24]. The strategy of utilizing heat stress to alleviate infection has been the basis of fomentation, infrared therapies, and saunas therapies to some positive effect [24]. The mechanism by which heat stress is leading to improved viral clearance in Fayoumi warrants further investigation to understand how an effective immune response is modulated during heat stress.

RNA-seq analysis has demonstrated a time-specific host response to NDV while under heat stress at the genome-wide level. Three time points (2, 6, and 10 dpi) at which the Harderian gland was profiled, captured the host innate, adaptive, and the transition between the responses throughout the course of NDV infection during heat stress. Distinct line and stage specific responses were observed (Fig. 3) based on the number of DEGs at the three different stages of infection. A single gene, protein kinase C delta (PRKCD), was the only gene that was differentially expressed across all three time points in Fayoumi. PRKCD encodes for a protein kinase that is a regulator of cell apoptosis and is often highly expressed in lymphoid tissue in humans [25]. A limited early response in both Fayoumi and Leghorn lines occurred at 2 dpi, although the Leghorn line had relatively more DEGs than Fayoumi. Leghorns eventually had a substantially stronger response at 6 dpi, while Fayoumi had a robust response at the later stages of infection under heat stress. In addition, very few differentially expressed genes overlapped between the lines at each time point suggesting a distinct, line-specific host response to NDV. A similar gene expression profile in terms of the number of DEGs observed in Harderian gland tissue was found in a parallel study in chickens under NDV infection only conducted at ISU by Deist et al. (Personal communication). However, the specific genes and signaling pathways identified in their study did not appear to have overlap with genes identified in this study. This suggests that understand the effects of both the combination of NDV and heat stress is critical to deepening our understanding of disease resistance to NDV in varying environments.

Several immune related DEGs (FADD, MAPK15, and GUCY1A2) in Fayoumi at 2 dpi, had been previously identified to be important in the host response to viral infection and the inflammatory response in cell lines [26–28]. FADD is of particular interest as it encodes for a protein that interfaces with cell surface receptors to mediate cell apoptotic signals that can help mitigate viral infections by disrupting mammalian cell replication [26]. Furthermore, knockout studies in mice suggest that this gene is crucial in promoting early T cell development and T cell activation [29]. Notably, FADD and MAPK15 were both up regulated (log_2_(fold change): 1.783 and 3.378) at 2 dpi in Fayoumi and down regulated (log_2_(fold change): − 1.854 and − 1.046) in Leghorns at 6 dpi. This suggests that following an infection by NDV, Fayoumis might have initiated T cell activation at 2 dpi, while this may have been inhibited in Leghorns at 6 dpi.

Interestingly, immune related pathways such as SAPK/JNK signaling and p38 MAPK signaling identified in Fayoumis at 2 dpi were also identified in Leghorns at 6 dpi, suggesting that these shared pathways were activated more rapidly in Fayoumi than in Leghorn. The most significant pathway identified within the Fayoumi line was related to Endothelin-1 signaling, which has previously has been identified as being important in the modulation of the inflammation response by immune cells [30]. Significantly fewer DEGs identified in Fayoumi compared to Leghorns at 6 dpi indicate that Fayoumis had not be experienced the infection as intensely as the Leghorn birds had and therefore required a lesser response to NDV infection. This was consistent with viral titer results measured through both the chicken lachrymal fluid and virus extracted reads from the Harderian gland at 6 dpi (Figs. 1 and 2).

At 10 dpi, there was a substantial increase in the overall number of DEGs in Fayoumi birds. Down regulation of genes such as GNB1, MAP3K1, CD247, and TNFRSF13B (log_2_(fold change): − 5.988, − 6.022, − 3.846, − 3.632) in immune-related pathways including SAPK/JNK signaling, Nur77 signaling in T lymphocytes, and B cell activation, suggest that Fayoumis may have begun to down modulate various processes associated with immunity in an attempt to restore homeostatic functions. However, treatment by line interaction analysis at 10 dpi showed that Leghorns had significantly higher activation of immune pathways such as NF-Kappa B activation by viruses, CD27 signaling, and B cell receptor signaling, which suggest that Leghorn birds may not have completely cleared the virus by 10 dpi. This prolonged immune response could be detrimental to the overall health of the host.

The observed global gene expression patterns observed by this study are wholly based on RNA-seq gene expression data. Functional assessment of candidate genes identified here (potentially by qPCR) may be needed in order to further understand the functional role of many of these genes that may be playing roles in regulating the chickens’ response to NDV while under heat stress. Ideally, we would desire to re-validate our findings through a separate cohort of samples, however due to the difficulty in accessing these samples, these experiments were not conducted at this study. In addition, the Ingenuity Pathway Analysis software uses primarily human and mouse biology curated database of pathways. The data was calculated using this background, however IPA supports the upload of genomic identifiers from a variety of species which includes chicken.

Only one pathway (GP6 signaling pathway), found in Leghorn birds at 6 dpi, was significantly enriched based on Z score when comparing the within line effects across all three time points (Fig. 7). The GP6 signaling pathway is involved with the activity of a protein in the immunoglobulin superfamily and is expressed in platelets and megakaryocytes [31]. GP6 proteins are also involved in collagen formation and function [31]. Previous research suggests that down regulation of collagen was associated with apoptosis, immune cell migration, and T cell activation [32–34]. Two genes, LAMA4 and COL4A1, were significantly up-regulated in this pathway and both these genes function in cell adhesion and play a role in regulating the migration cells such as neutrophils in mammals [35, 36]. One study revealed that gene expression of collagen genes in the trachea were lower in resistant birds infected with infectious bronchitis virus [29]. In addition, a parallel study at ISU investigating the host response to NDV infection without heat treatment found significantly lower expression of these genes in Fayoumi birds at 2 dpi compared to Leghorn birds [11]. Collectively, activation of the GP6 signaling pathway, through increase collagen gene expression, at 6 dpi could be partially responsible for the Leghorn lines susceptible phenotype.

The interaction analysis of treatment by genetic line would provide further insights into the resistant and susceptible phenotypes of the Fayoumi and Leghorn lines. While only one signaling pathway was identified in the within line contrasts, a number of significant signaling pathways were enriched from the interaction analysis of treatment by genetic line. The majority of enriched pathways were found at 2 dpi and 10 dpi, with 6 dpi have the least enrichment. This suggests that distinct signaling pathways were enriched between the two lines but were undetectable when only investigating for within line effects.

## Conclusion

Transcriptome analysis of two unique genetic lines has helped identify line and time specific gene expression patterns related to NDV infection under the effects of heat stress. The Harderian gland demonstrated very unique response profiles between the two genetic lines, both in the number of DEGs identified, and the pathways that were enriched within each line. The overall lower viral titer levels, detectable viral transcripts, and increased viral clearance rate observed in Fayoumi suggest that the birds were effectively responding to NDV infection while under heat stress than the Leghorn birds. Fayoumis appeared to have activated immune related pathways that were in line with a more robust immune response at an earlier time point, while Leghorns were clearly responding up to 6 dpi. Additional candidate genes such as FADD, MAPK11, and MAPK15 that were identified as critically important genes in regulating the chickens’ response to NDV infection during abiotic stress. Future investigations on these novel candidate genes and signaling pathways will allow us to elucidate the different regulatory mechanisms of disease resistance to NDV under heat stress. More importantly, these efforts will lay solid foundation on achieving the overall goal of Genomics to Improve Poultry Innovation Lab: improve poultry production by genetically enhancing disease resistance to NDV infection in Africa.

## Methods

### Experimental populations and design

The experimental design of this study has been previously described by Wang et al. [20]. Fayoumi (M15.2) and Leghorn (GHs 6) chicken lines from Iowa State University poultry farm (Ames, IA) were used in this study. On day of hatch, 56 Fayoumi and 55 Leghorn chicks were transported from Iowa State University to Davis, CA. Upon arrival, the chicks were housed in temperature and humidity-controlled chambers at the biosafety level 2 animal facility at the University of California, Davis. Twenty-five individuals from each genetic line were randomly selected and housed in a separate chamber to be used as the control group. From day 1 to day 13 both groups were reared at 29.4 °C and 60% humidity. At 14 days of age, the experimental group was exposed to 38 °C for 4 h, then decreased to 35 °C and maintained at this temperature until the conclusion of the trial. The control group was maintained at 25 °C. On day 21 the heat treated birds were inoculated with 200 μL 10^7^ EID_50_ of the La Sota strain of NDV through both ocular and nasal passages. The control group was mock inoculated with 200 μL of 1X phosphate-buffered saline (PBS). At 2, 6, and 10 days post-infection (dpi), 4 birds per treatment group per genetic line were randomly selected and euthanized with CO_2_ and Harderian gland tissue was harvested then, quickly placed into RNA*later* (ThermoFisher Cat#AM7024) and kept at − 80 °C. The experiment’s procedures were performed according to the guidelines approved by the Institutional Animal Care and Use Committee at the University of California, Davis (IACUC #17853).

### Viral titer measurement

Viral RNA was extracted from chicken lachrymal fluid at 2 and 6 dpi from both control and infected groups and extracted using the MagMAX-96 viral RNA isolation kit (Life Technologies Cat#AMB18365). Quantification of the virus was conducted using qRT-PCR using the TaqMan Newcastle Disease Virus Real-Time PCR kit (Life Technologies Cat#44006874) and measured on the ABI 7500 fast Real-Time PCR system (Thermofisher Cat#4351107, Carlsbad, CA). A standard curve was generated from a log copy number dilution of the virus from 10^5^ to 10^2^ EID_50_ and used to calculate the viral titer in tears. Viral clearance was calculated as the difference in viral log copy number from 2 to 6 dpi divided by the viral log copy number at 2 dpi.

### Anti-NDV antibody measurement

Serum samples were extracted from whole blood collected at pre-challenge (day 20) and 10 dpi (day 31). Antibodies in the serum were then measured using the IDEXX NDV ELISA kit for chickens (IDEXX Laboratories Cat#99–09263). The Sample to Positive ratio (S/P) was calculated by the average absorbance of each sample divided by the recorded measurement of the provided kit control.

### RNA-isolation and library construction

Total RNA was extracted from the Harderian gland of four individuals per treatment and genetic line for each of the three time points. The Harderian gland was homogenized in ice cold TRIzol (ThermoFisher Cat#15596026) and processed using a standard phenol:chloroform method and precipitated in 100% ethanol. The RNA pellet was then dissolved into water and treated with DNase I (ThermoFisher Cat#EN0521). Strand specific RNA library preparation was prepared exactly as stated in the NEBNext Ultra Directional RNA Library Prep Kit for Illumina (NEB Cat#E7420S). Library validation and quantification was done using the Agilent Bioanalyzer High Sensitivity Kit (Agilent Cat#5067–4626) and Qubit dsDNA HS Assay kit (ThermoFisher Cat#Q32854). The 100 base pair, paired-end sequencing was performed on the Illumina HiSeq2500 system with a minimum sequencing depth of 30 million reads. Sequence data have been submitted through the Sequence Read Archive (https://www.ncbi.nlm.nih.gov/sra/) under accession number: SRP135507.

### NDV viral genome alignment

Unaligned transcript reads from the chicken genome of the treated individuals were aligned to the NDV La Sota reference genome using the Burrows-Wheeler Aligner (BWA) [37].Default settings were used and gene counts from the NDV genome were calculated using HTSeq [38].

### Data analysis

Analysis of viral titer and anti-NDV S/P ratio differences between the two lines was analyzed using a least squares regression analysis with the main effects including line, day, and line by day. A Student’s T-test was used to determine significance, with a *p* < 0.05 considered statistically significant between comparisons. Statistical analysis and visualization of the viral titers was performed using the statistical data analysis software R with standard packages [39].

Four major factors were included for analysis: condition (treated, non-treated), line (Leghorn, Fayoumi), sex (male, female), and time point (2, 6, and 10 dpi). Data at each time point consisted of 16 individuals, 4 per treatment and genetic line. Raw reads from RNA-seq were trimmed using FastQC [40] to remove duplicates, reads with base quality scores < 30, and adapter contamination. These reads were then aligned using Tophat2 [41] to the galGal5 reference genome and Ensembl annotation using default settings and a summary of the alignment statistics can be found in Table 1. Gene counts were calculated using HTSeq and differential gene analysis was done using edgeR [42]. The statistical model design included the effects of line, condition, sex, and time point, along with the interactions between condition and line. In addition, in order to identify genes that were differentially expressed between genetic lines in response to treatment and, therefore, potentially associated with disease resistance to NDV, the interaction between condition and genetic line was included. Genes were identified as differentially expressed (DEGs) if they had a false discovery rate (FDR) < 0.05, and an average transcript count > 10. Pathway analysis using the DEGs of between line contrasts and the interaction effect was performed using Qiagen’s Ingenuity Pathway Analysis software [43]. Z-score cutoff of |z| > 1 identified significantly up or down regulated pathways [43].

**Table 1:** Summary statistics of RNA-Seq alignment

		Average number of reads mapped (bp)	Average % of reads mapped
Fayoumi	Treated	21,285,421	75.59
	Non-Treated	24,270,472	76.70
Leghorn	Treated	21,092,774	72.31
	Non-Treated	18,515,551	70.92

## Additional file

Additional file 1: List of DEGs produced by edgeR for all contrasts presented in this study. (XLS 173 kb)

## Abbreviations

DEG: Differentially expressed gene
dpi: Days post-infection
FDR: False Discovery Rate
LFC: Log_2_(fold change)
NDV: Newcastle disease virus
